# External validation of the bellmunt risk score for survival prediction in mCRPC patients undergoing [^177^Lu]Lu-PSMA-617 therapy

**DOI:** 10.1007/s00259-026-08169-7

**Published:** 2026-09-08

**Authors:** Thomas Büttner, Mike Wenzel, Philipp Mandel, Paul Schmitt, Christian Hoffmann, Milka Marinova, Markus Essler, Felix K. H. Chun, Manuel Ritter, Daniel Groener, Philipp Krausewitz

**Affiliations:** 1 Department of Urology and Pediatric Urology, University Hospital Bonn, Venusberg-Campus 1, Bonn, 53127 Germany; 2 Department of Urology, University Hospital Frankfurt, Frankfurt am Main, Germany; 3 Martini-Klinik Prostate Cancer Center, University Hospital Hamburg- Eppendorf, Hamburg, Germany; 4 Department of Nuclear Medicine, University Hospital Bonn, Bonn, Germany; 5 Department of Nuclear Medicine, Clinic for Radiology and Nuclear Medicine, University Hospital Frankfurt, Frankfurt am Main, Germany

**Keywords:** mCRPC, Lutetium, PSMA, Bellmunt risk score, Prediction, Survival

## Abstract

**Purpose:**

[^177^Lu]Lu-PSMA-617 is a standard-of-care therapy for metastatic castration-resistant prostate cancer (mCRPC). Given the heterogeneous clinical responses, accessible prognostic tools are needed. We recently demonstrated that the Bellmunt Risk Score (BRS)—comprising ECOG performance status >0, hemoglobin <10 g/dL, and the presence of liver metastases—predicts overall survival (OS) in a primary mCRPC cohort. This study aims to externally validate the BRS in an independent cohort.

**Methods:**

We retrospectively evaluated 243 mCRPC patients treated with [^177^Lu]Lu-PSMA-617 at an independent tertiary center. The BRS was calculated at baseline (range 0-3). Endpoints included OS analyzed via Kaplan-Meier estimators, multivariable Cox regression, Harrell’s C-index, and time-dependent area under the curve (tAUC).

**Results:**

The cohort was distributed into BRS 0 (17.6%), BRS 1 (52.7%), BRS 2 (26.1%), and BRS 3 (2.9%). Kaplan-Meier analysis revealed significant risk stratification, with an estimated median OS of 24.0, 15.8, 11.2, and 8.7 months for BRS 0, 1, 2, and 3, respectively (*p* < 0.001). In univariable analysis, BRS significantly predicted survival. In multivariable Cox regression adjusted for the number of prior treatment lines, BRS 2 (HR 3.04, *p* < 0.001) and BRS 3 (HR 4.76, *p* = 0.002) remained independent predictors of shortened OS. The BRS demonstrated robust predictive accuracy, achieving a tAUC of 71.3% for 1-year OS.

**Conclusion:**

The BRS is a pragmatic, robust, and easily calculable prognostic tool for mCRPC patients undergoing [^177^Lu]Lu-PSMA-617 therapy, successfully validated in an independent external cohort.

**Supplementary Information:**

The online version contains supplementary material available at https://doi.org/10.1007/s00259-026-08169-7.

## Introduction

Despite the paradigm-shifting introduction of [^177^Lu]Lu-PSMA-617 radioligand therapy (RLT) for metastatic castration-resistant prostate cancer (mCRPC) following the pivotal VISION phase III trial [1], individual survival benefits remain highly heterogeneous. An accurate prognostic assessment is crucial to properly weigh the potential benefits of treatment against its burden.

While several nomograms combining advanced imaging parameters, genomics, and clinical data have been developed ADDIN EN.CITE [2, 3], their routine implementation is often hindered by complexity and the lack of readily available data in daily clinical practice. Consequently, there is an unmet clinical need for simple, universally accessible prognostic scoring systems.

The Bellmunt Risk Score (BRS), originally established for metastatic urothelial carcinoma, stratifies patients using three simple, universally available binary parameters: impaired performance status (ECOG PS >0), anaemia (hemoglobin <10 g/dL), and the presence of liver metastases [4]. In a recent primary analysis, we demonstrated that the BRS effectively translates to the mCRPC population, predicting overall survival (OS) in patients receiving [^177^Lu]Lu-PSMA-617 [5].

To confirm generalizability and clinical robustness, external validation in an independent patient population is mandatory. Therefore, the present study aimed to externally validate the BRS for OS prediction in a distinct, independent, real-world cohort of mCRPC patients undergoing [^177^Lu]Lu-PSMA-617 RLT.

## Methods

### Study population

This retrospective observational study included patients with confirmed PSMA-positive mCRPC who received at least one cycle of [^177^Lu]Lu-PSMA-617 therapy at an independent tertiary high-volume center (University Hospital Frankfurt). There was no overlap in patient populations with the original development cohort, and data extraction for the validation cohort was performed by investigators not involved in the original cohort’s data collection. Institutional treatment pathways and [^177^Lu]Lu-PSMA-617 application protocols were maintained independently.

Further inclusion criteria were complete information on baseline hemoglobin value, ECOG PS, and imaging data. Patients with missing prerequisite variables were excluded prior to the final survival analysis without imputation.

### Bellmunt risk score and endpoints

The BRS was determined at baseline immediately prior to the first RLT cycle (maximum -28 days). Patients were assigned one point for each of the following risk factors: ECOG PS >0, hemoglobin <10 g/dL, and the presence of liver metastases, resulting in a score ranging from 0 to 3. The presence of liver metastases was determined by morphology and/or focal PSMA uptake in baseline pre-therapeutic imaging, primarily utilizing [^68^Ga]Ga-PSMA-11 or [^18^F]PSMA-1007 PET/CT and relying on local institutional interpretation; no central review was conducted. The primary endpoint was OS, defined as the time from the first RLT administration to death from any cause or the date of last follow-up.

### Statistical analysis

Baseline characteristics were reported descriptively. Survival curves were estimated using the Kaplan-Meier method and compared via the log-rank test. Uni- and multivariable Cox proportional hazards regression models were utilized, adjusting for the number of prior therapy lines as a potential confounder of OS. Firth’s penalized likelihood Cox regression was used for sensitivity analysis in small sample sizes. Predictive accuracy was assessed using Harrell’s C-Index and time-dependent Area Under the ROC Curve (tAUC) for 6-, 12-, and 24-month survival. Model calibration for 12-month overall survival was assessed using calibration plots with 200 bootstrap resamples, quantifying mean and maximum absolute errors. Analyses were performed using RStudio 2026.01.0+392 with a two-sided significance level of *p* < 0.05.

## Results

### Baseline characteristics

A total of 243 patients were included in the validation cohort. The median baseline hemoglobin was 11.6 g/dL (range 6.0–15.8), with 23.9% of patients presenting with anemia <10 g/dL. An impaired functional status (ECOG PS >0) was present in 80.2% of cases, and 10.3% had liver metastases. Over a median follow-up of 17.7 months (95% CI: 15.1–30.7), a total of 111 deaths (45.7%) occurred. Following BRS calculation, 43 patients (17.7%) exhibited zero risk factors (BRS 0), 129 (53.1%) had a BRS of 1, 64 (26.3%) a BRS of 2, and 7 (2.9%) presented with a BRS of 3. Most patients were treated in a considerably advanced setting, with 9.9%, 22.6%, and 39.1% receiving RLT in the 1st, 2nd, and 3rd mCRPC treatment line, respectively, and 28.4% in even later lines. Detailed patient characteristics are provided in Table 1.

**Table 1:** Baseline characteristics of the study cohort

Parameter	Overall Cohort *n* = 243
Age (years)	
Mean (SD)	72.5 (8.7)
Median [Range]	73.7 [41.0, 92.0]
Missing	2 (0.8%)
Hemoglobin (g/dL)	
Mean (SD)	11.4 (1.8)
Median [Range]	11.6 [6.0, 15.8]
Anemia < 10 g/dL, n (%)	58 (23.9%)
ECOG Performance Status, n (%)	
0	48 (19.8%)
1	148 (60.9%)
2	38 (15.6%)
3+	9 (3.7%)
Liver Metastases	
Absent	218 (89.7%)
Present	25 (10.3%)
Prior mCRPC Treatment Lines, n (%)	
0	24 (9.9%)
1	55 (22.6%)
2	95 (39.1%)
3+	69 (28.4%)
Bellmunt Risk Score	
0	43 (17.7%)
1	129 (53.1%)
2	64 (26.3%)
3	7 (2.9%)

*ECOG* Eastern Cooperative Oncology Group Performance Status, *SD* standard deviation

### Validation of the BRS for overall survival

**Fig. 1:**
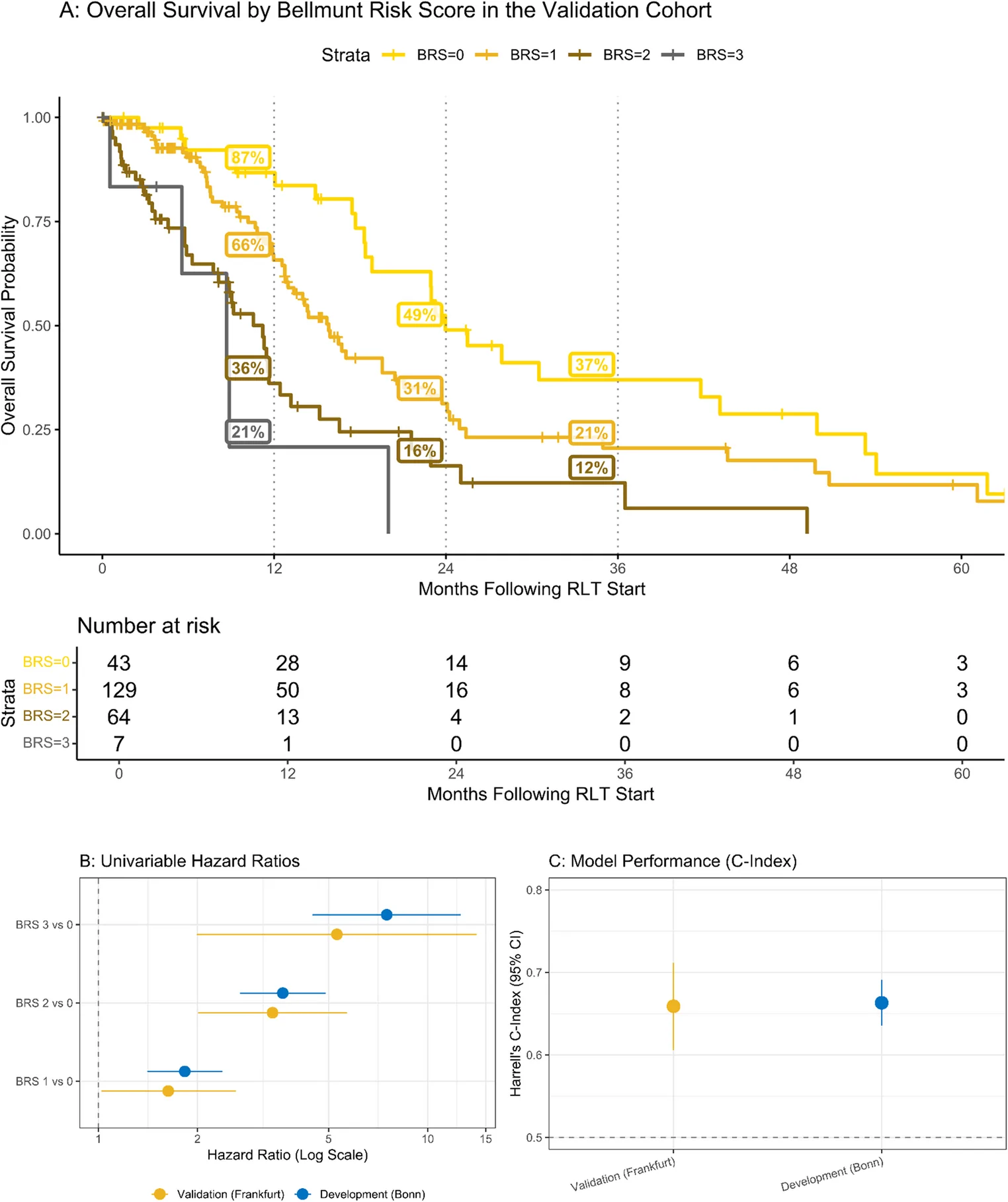
(**A**) Kaplan-Meier estimates for Overall Survival (OS) according to the Bellmunt Risk Score in the external validation cohort with landmark 1-year, 2-year and 3-year OS rates. (**B**) Forest plot demonstrating univariable Hazard Ratios (HR) and 95% Confidence Intervals for mortality according to BRS groups, directly comparing the external validation cohort (Frankfurt) with the primary development cohort [5]. (**C**) Point-range plot displaying Harrell’s Concordance-Index (C-Index) with 95% Confidence Intervals, comparing the overall prognostic accuracy of the BRS between the validation and development cohorts

The BRS successfully stratified patients into distinct prognostic groups. In the Kaplan-Meier analysis, patients with a BRS of 0 demonstrated a median OS of 24.0 months (95% CI: 18.8–49.9). Median OS decreased consecutively with each additional risk factor: 15.8 months (95% CI: 13.0–21.1) for BRS 1, 11.2 months (95% CI: 8.1–13.2) for BRS 2, and 8.7 months (95% CI: 5.6–NA) for BRS 3 (Log-rank *p* < 0.0001; Fig. 1A).

In univariable Cox regression analysis, a higher BRS was significantly associated with a higher risk of mortality. Compared to the low-risk BRS 0 group, the hazard ratio (HR) for mortality was 1.63 for BRS 1 (95% CI: 1.02–2.62, *p* = 0.040), 3.38 for BRS 2 (95% CI: 2.01–5.69, *p* < 0.001), and 5.30 for BRS 3 (95% CI: 1.99–14.10, *p* < 0.001). Importantly, these HRs aligned with the prognostic effects observed in the primary development cohort (Fig. 1B).

In multivariable Cox regression analysis—adjusted for the number of prior mCRPC treatment lines—the BRS remained a predictor of survival. Compared to the low-risk BRS 0 group, the HR for mortality was 1.49 for BRS 1 (95% CI: 0.92–2.43, *p* = 0.107), 3.04 for BRS 2 (95% CI: 1.77–5.24, *p* < 0.001), and 4.76 for BRS 3 (95% CI: 1.76–12.86, *p* = 0.002). To address the small sample size in the BRS 3 subgroup, sensitivity analysis confirmed the stability of the estimates (Firth HR for BRS 3: 5.10, 95% CI: 1.77–12.42, *p*=0.004).

### Predictive performance

The multivariable model incorporating the BRS and treatment line achieved a Harrell’s C-index of 0.659 (95% CI: 0.606 – 0.712). This predictive performance proved to be comparable to the prognostic accuracy of the BRS in the original development cohort (Fig. 1C). When evaluating the dynamic longitudinal accuracy of the BRS alone via time-dependent ROC analysis, the score yielded tAUCs of 69.3% (95% CI: 59.5–79.1) for 6-month, 71.3% (95% CI: 64.0–78.5) for 12-month, and 67.5% (95% CI: 58.2–76.8) for 24-month overall survival prediction. As shown in the calibration plot (Supplementary Fig. S1), the model demonstrated excellent 12-month calibration (mean absolute error: 0.027; maximum absolute error: 0.059).

## Discussion

The need for practical, real-world applicable prognostic stratification in mCRPC remains high, particularly as the indications for [^177^Lu]Lu-PSMA-617 expand into earlier treatment lines [6]. In this study, we successfully validated the Bellmunt Risk Score—a simple tool requiring only ECOG PS, hemoglobin, and imaging of the liver—in an independent, external cohort of 243 patients.

Our findings demonstrate a clear stepwise prognostic effect: each additional BRS risk factor resulted in a clinically meaningful reduction in median overall survival, dropping from 24.0 months in patients with no risk factors to 8.7 months in patients with three risk factors. Since the primary objective of this study was to validate the prognostic utility of BRS, adjustment of the multivariable analysis was intentionally limited to the number of prior mCRPC treatment lines. While this confirmed its robust nature across different stages of mCRPC, the inclusion of biochemical markers (such as further laboratory values) or advanced volumetric imaging parameters had to be omitted, resulting in uncertainties regarding confounding or complementary effects. However, this avoids model overfitting and preserves the BRS’s identity as a strictly clinical, standalone, bedside-ready tool that does not rely on variables that may be inconsistently available across different real-world practices: While more complex nomograms incorporating quantitative PSMA-PET volume metrics or biochemical panels theoretically offer superior prognostic accuracy [2, 7], they require specialized software or incur additional laboratory costs. In contrast, the primary clinical advantage of the BRS lies in its absolute simplicity, thereby serving as a highly pragmatic tool for immediate risk stratification and shared decision-making.

Notably, the external validation cohort evaluated here showed slight demographic variations compared to our primary development cohort. Patients in the present cohort had a somewhat more favorable overall prognosis (e.g., median OS for BRS 0 was 24.0 months vs. ~18 months in the primary cohort, and liver metastases were less frequent at 10.2% vs. 17.6%). Despite these differences in baseline characteristics and institutional treatment pathways, the BRS maintained discriminatory power and predictive accuracy especially for a semi-quantitative scoring system (C-index 0.659, 12-month tAUC >70%). These metrices, comparative to those in the development cohort, underscore the generalizability of the score, confirming that it performs reliably across varying real-world patient populations.

Limitations of this study include its retrospective nature and the absence of central imaging review. Furthermore, it must be emphasized that the highest risk category (BRS 3) contained a very small number of patients (*n*=7). This small sample size substantially limits the reliability and generalizability of the survival estimates and hazard ratio calculations for this specific subgroup, necessitating a cautious interpretation of the outcomes for patients presenting with all three risk factors. Finally, the multivariable analysis was limited in its adjustment for other established prognostic factors, such as baseline PSA, LDH, alkaline phosphatase, and quantitative metastatic burden. The omission of these covariates may introduce residual confounding, and the potential impact of these unmeasured variables on overall survival outcomes cannot be entirely excluded. However, the strong overall statistical significance and the consistency with primary findings partly mitigate these limitations.

## Conclusion

The Bellmunt Risk Score is a robust, pragmatic, and independent prognostic tool for predicting overall survival in mCRPC patients treated with [^177^Lu]Lu-PSMA-617. Having been successfully validated in an independent external cohort, it allows for rapid, bedside risk stratification. Importantly, the BRS is intended to serve as a complementary prognostic stratification tool to guide patient expectations, rather than a replacement for comprehensive clinical and imaging assessment in individual treatment decision-making.

## Supplementary Information

Below is the link to the electronic supplementary material.

Supplementary Material 1 (DOCX 37.2 KB)

## Data Availability

The datasets generated during and analysed during the current study are available from the corresponding author on reasonable request.
