# Supplementary material for: External validation of the bellmunt risk score for survival prediction in mCRPC patients undergoing [177Lu]Lu-PSMA-617 therapy
Source: Eur J Nucl Med Mol Imaging. 2026 Sep 8;53(13):8514–8. doi: 10.1007/s00259-026-08169-7 (PMC13727873; doi:10.1007/s00259-026-08169-7)
Supplement: Supplementary file 1 [file 259_2026_8169_MOESM1_ESM.docx]

**Supplementary Material to**

**External Validation of the Bellmunt Risk Score for Survival Prediction in mCRPC Patients Undergoing [^177^Lu]Lu-PSMA-617 Therapy**

**Supplementary Figure S1:** Calibration plot assessing the multivariable model’s performance for 12-month overall survival. The x-axis represents the model-predicted 12-month survival probability, and the y-axis displays the actual observed survival probability (Kaplan-Meier estimates). The faint diagonal line indicates perfect ideal calibration. The solid line with points depicts the model's actual performance, internally validated using 200 bootstrap resamples, demonstrating strong agreement between predicted and observed outcomes (mean absolute error: 0.027; maximum absolute error: 0.059).
